# A Case of Villoglandular Papillary Adenocarcinoma of the Uterine Cervix Diagnosed during Early Pregnancy Followed by Successful Term Delivery

**DOI:** 10.1155/2010/314547

**Published:** 2010-06-10

**Authors:** Noriyuki Takai, Chihiro Hayashita, Satoru Nakamura, Hisashi Narahara, Hideo Matsumoto

**Affiliations:** ^1^Department of Obstetrics and Gynecology, Oita University Faculty of Medicine, 1-1 Idaigaoka, Hasama-machi, Yufu-shi, Oita 879-5593, Japan; ^2^Department of Obstetrics and Gynecology, Oita Prefectural Hospital, 476 Bu-nyo, Oita 870-8511, Japan

## Abstract

Villoglandular papillary adenocarcinoma (VPA) is a very rare subtype of adenocarcinoma of the uterine cervix, but a well-recognized variant of cervical adenocarcinoma with a favorable prognosis and generally occurring in women of child-bearing age. Only five cases of VPA and pregnancy have been reported. Herein, we report a case of VPA diagnosed during early pregnancy and managed successfully with conservative measures; our patient delivered a healthy baby in full term. A successful pregnancy can be completed in patients with VPA without lymph-vascular invasion, when treated conservatively. This management is particularly desirable in young women to preserve reproductive capability.

## 1. Introduction

The incidence of cervical adenocarcinoma is on the rise over the last decades. Villoglandular papillary adenocarcinoma (VPA) is a very rare subtype of adenocarcinoma of the uterine cervix. The true incidence of this form of adenocarcinoma is unknown. The classical histologic appearance of this entity is a surface papillary component of variable thickness with papillae that are usually tall and thin, but occasionally short and broad, with a fibrous stromal core. The tumor cells should have no more than mild-to-moderate nuclear atypicality and scattered mitotic figures. It affects a younger age group and has an excellent prognosis as compared to other endocervical adenocarcinomas [1, 2]. To our knowledge, only five cases of VPA associated with pregnancies have been reported in the literature [3–7]. In two cases, the patients were diagnosed VPA during pregnancy followed by conservative treatment and delivered healthy children [5, 7]. We report here a successful term pregnancy with stage IB VPA of the cervix diagnosed during early pregnancy. 

## 2. Case Report

**Figure 1:**
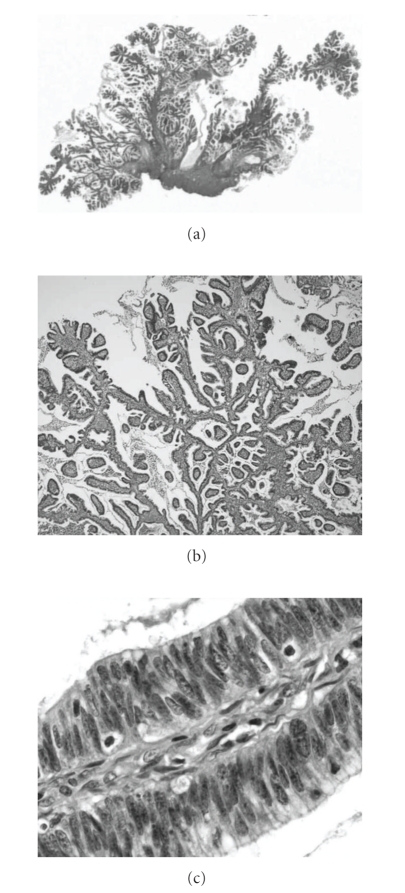
Typical histological patterns for villoglandular papillary adenocarcinoma of the cervix. (a) Tumor displaying thin and tall, well-formed papillary structures (hematoxylin and eosin, original magnification, x4), (b) Higher magnification of (a). Large glandular and papillary structures with broad stroma (hematoxylin and eosin, original magnification, x40), (c) Higher magnification of (b) (hematoxylin and eosin, original magnification, x200).

A 28-year-old Japanese woman, gravida 1, para 1, was admitted with atypical genital bleeding and underwent polypectomy of the uterine cervix at 9 weeks' gestation. The 1-cm resected polyp was pathologically diagnosed as VPA (Figure 1). The tumor is purely exophytic without invasion of the underlying stroma and lymphvascular involvement. Hence, she was referred to our hospital. As the polyp had no lymph capillary space invasion, we performed conization at 16 weeks' gestation when the patient decided to continue her pregnancy. The depth of surgical specimen was 1 cm and width of that was 3 cm diameter. No cancer cells were identified in the resected specimens. The final diagnosis was FIGO stage IB1. She delivered a healthy 2,946 g newborn vaginally at term 38 weeks, and she has been free of the disease for 44 months.

## 3. Discussion

We report an extremely rare case who was diagnosed VPA in the 1st trimester and was managed conservatively throughout the pregnancy with successful results for both the mother and the baby. VPA of the uterine cervix is a rare form of cervical adenocarcinoma first described by Young and Scully in 1989 [1]. They found that this tumor has an excellent prognosis and suggested conization as a potential treatment for patients of childbearing age [1]. Conservative management of cervical VPA is considered to be a significant challenge; however, the English literature concerning treatment of VPA diagnosed during pregnancy is sparse. So far, over 115 cases of cervical VPAs have been reported worldwide; of these only nine metastases and two deaths were reported [7–11]. These few cases show an apparent discrepancy from the excellent prognosis of VPA described originally by Young, Scully and others [1, 12]. In 30% of cases, VPA is associated with other forms of invasive cancer [1–4, 6], which may have an important impact on the prognosis. Young and Scully therefore reserve the term VPA for tumors in which the villoglandular pattern is the exclusive or almost exclusive one. It has been suggested that in cases of superficial VPA diagnosed in young patients, unassociated with another type of cervical tumor and without lymph vascular invasion, less radical treatment may be suitable since these cases present a favorable outcome [12]. However, since the knowledge of the biologic spectrum of VPA appears to be evolving, a close follow up should be pursued in VPA patients managed conservatively [13].

Young and Scully recommended careful inspection of the histological specimen and if the villoglandular component is the exclusive or almost exclusive pattern then a diagnosis of VPA can be ascribed [1]. Other papillary adenocarcinomas can present a difficulty in diagnosis. Serous papillary adenocarcinomas of the cervix have finer, more irregular and more cellular papillae than VPA. The clear cell papillary adenocarcinomas of the cervix are characterized by marked cytological atypia, high mitotic activity and occasionally the presence of psammoma bodies. VPA should be distinguished from endocervical adenocarcinoma with a minor villoglandular component. The rare adenosarcoma and adenoma malignum should also be considered in the differential diagnosis of VPA [8].

Pregnancy associated with VPA of the cervix has been reported in only five cases [3–7]. In two cases, successful pregnancies were achieved following a conservative treatment for VPA [3, 4]. Three additional cases were diagnosed during pregnancy (Table 1); the first case, which was diagnosed during the 20th week of gestation, was conservatively followed until the 32nd week of gestation, when a caesarean radical hysterectomy was performed [5], the second case ended with an early induced abortion (8 weeks of gestation) followed by a radical hysterectomy [6], and the third case, which was diagnosed during the 13th week of gestation, was conservatively followed until the 37th week of gestation, when a caesarean radical hysterectomy was performed [7]. In the case whose pregnancy was terminated (8 weeks of gestation) followed by a radical hysterectomy, the patient underwent second, third and fourth laparotomies because of recurrent pelvic masses. At the end of five years follow-up period, she died because of the complication of recurrent tumor [6]. Bouman et al. reported three cases of VPA, and two of these are malicious because they have other histological features (the first case showed well-differentiated adenocarcinoma with abundant squamous differentiation, and the second case has well to moderately differentiated papillary adenocarcinoma) [14]. These authors recommend the attitude, “Beware of a wolf in sheep's clothing”, in relation to VPA [14]. However, we can not discuss the clinical outcome in pregnant patients, because there is no report of VPA accompanied with other histological features or extended tumor invasion in pregnant patients.

In conclusion, despite the limited experience of cervical VPA diagnosed during pregnancy, conservative treatment can be successfully achieved in selected patients after a thorough evaluation of the depth of invasion, the lymph vascular involvement, and the association of other carcinoma histologies in conjunction with the VPA (i.e. adenocarcinoma or squamous cell carcinoma).

## Figures and Tables

**Table 1:** Profile of patients with villoglandular papillary adenocarcinoma diagnosed in pregnancy.

Reference	Present case	[5]	[6]	[7]
Age	28	22	28	31
Gravida	1	3	3	2
Para	1	2	2	1
Gestational age at diagnosis (weeks)	9	20	8	13
Gestational age at delivery (weeks)	38	32	8	37
Mode of delivery	VD	C/S	Termination	C/S
Macroscopic feature	Polypoid	Polypoid	Polypoid	Polypoid
FIGO stage	IB1	IB2	IB1	IB1
Treatment	Conization	RH	RH	RH
LCSI	—	—	—	—
Lymph node metastasis	Not examined	—	Not examined	—
Follow-up (months)	38	14	60	18
Outcome	NED	NED	DOD	NED

VD: vaginal delivery; C/S: cesarean section; FIGO: Fédération Internatinale de Gynécologie et d'Obstétrique; RH: radical hysterectomy; LCSI: lymph capillary space invasion; NED: no evidence of disease; DOD: dead of disease.
